# Reviews of Dental Literature

**Published:** 1905-08

**Authors:** 


### Reviews of Dental Literature.

Hand Sterilization.—In carrying out hand sterilization, mechanical and chemical methods are employed.

(1) *Mechanical.*—Careful use of a stiff nail-brush with soap and hot water removes many bacteria and detaches superficial epidermal cells and grease with contained organisms. The time required and the injurious effect on the hands render it beyond the limit of practicability to produce efficient sterilization by this method alone.

(2) *Chemical.*—Ordinary operating-room methods are to a considerable extent inefficacious in rendering the hands sterile. The fact that cultures of a staphylococcus could be obtained from an inoculated silk thread after thirty minutes in a watery solution of corrosive sublimate (1 in 1000) speaks for the weakness of this popular preparation. Absolute alcohol has but a slight germicidal power, but the diluted fluid (seventy per cent.) has a far greater action than a watery solution of bichloride or biniodide of mercury (1 in 1000) or a saturated solution of permanganate of potash. Prolonged use of alcohol, however, will cause pain, roughen the skin, and may produce eczema.

The writer has held that the ideal cleansing agent must be a solution capable of dissolving fatty matter and of penetrating the epidermis, strongly germicidal, rapid in action, and non-injurious to the skin. After long experimentations the writer suggests the unpurified clove oil as an approach to the ideal. After five minutes' scrubbing with soap and hot water the skin should be dried with a sterile towel and rubbed for one minute with alcohol to remove any remaining moisture. Clove oil is then rubbed into the skin for five minutes and afterwards washed off with alcohol. A slight burning sensation may result, but the skin is not injured. The hands should then be covered with dry sterile rubber gloves. Wet gloves macerate the skin, which may readily yield organisms which have not been destroyed through any undetected hole in the glove. The use of various "hand coatings," wax paraffins, and rubber solutions are not to be recommended, as they are prone to crack and peel off in long operations.—J. C. WEBSTER (*American Journal of Obstetrics*, April, 1905), *Cyclopedia of Practical Medicine.*

RESEARCHES IN HÆMOPHILIA.—The writer had the rare luck to observe four typical cases of hæmophilia and to study the peculiarities of the blood in this strange disorder. According to some, the condition is due to high blood-pressure, but this is improbable, since diseases commonly associated with high blood-pressure, such as chronic nephritis, do not usually run with hæmophilia symptoms. In the author's case, the figures obtained with the Riva-Rocci instrument were normal or below normal. Microscopical examination of the blood showed only a moderate, relative diminution of polynuclear leucocytes, with relative increase of lymphocytes. The absolute number of leucocytes was normal or diminished. In two cases the platelets were also counted repeatedly, but their number was never above normal. The alkalinity of the blood, the dry residue of the serum, the depression of the freezing point, and the amount of fibrin in the blood were not altered. The time of coagulation was estimated most carefully, and it was found that in the intervals between the hemorrhages clotting was much delayed, but normal, or even hastened, during severe bleeding. The following new method was employed: A column of blood, about one centimetre high, is allowed to flow into a capilary pipette one to two millimetres in diameter. An absolutely clean, white strand of horsehair is then passed into the blood, and drawn out a short distance every half to one minute. If the hair has been carefully deprived of all grease, no blood will adhere to it at first, but as soon as coagulation has set in, the withdrawn section will no longer appear white, but red. The rapid clotting during bleeding, despite continued hemorrhage, is probably due to an abnormal quality of the vessel walls. Under normal conditions, the latter probably furnish certain substances necessary for the production of fibrin-ferment (thrombo-kinase) locally at the site of injury, so that a clot will soon obstruct the opening in the vessel. During hemophilia, the torn edges of the vessel do not supply the blood with this substance, hence no local clot forms. The imperfect clotting during the intervals is due to a similar deficiency on the part of the blood-cells and the hæmatopoietic apparatus. Chemical changes in the vessel walls will also explain the occurrence of spontaneous hemorrhages and the reported cases of hæmophilia of single organs (Senator's renal hæmophilia). Very little can be done for the disease, except to improve the general constitution. The local hemorrhages are best controlled with compression, gelatin, and adrenalin, but the latter two drugs should never be injected subcutaneously. It is not likely that the local application of thrombo-kinase will do much good. There is as yet no drug from which good results can be expected on internal administration.—H. Sahli (*Zeitschrift für klinische Medicin,* Bd. lxv., Nu. 3 und 4; *Medical News,* May 6, 1905), *Cyclopedia of Practical Medicine.*

Parotid Gland.—The function of the parotid gland is the subject of a scientific research by the writer, who found that the secretion of the gland varies in amount and quality according to the stimulus given by contact with various substances in the mouth. The amount of secretion of the parotid varies as the square root of the amount of stimulant applied to the mouth. Chewing has much to do with the rapidity of the secretion from the parotid, and the saliva is not only more abundant, but more viscid, when mastication is vigorous and prolonged. When food is chewed on one side, the corresponding parotid gland works more than the opposite gland, while when chewing goes on on both sides both glands work equally. The alkalinity of the saliva is in proportion to the amount of ash therein. As the amount of ash increases, and as the rapidity of the secretion is enhanced, the alkalinity grows more marked. The digestive power of the saliva is proportionate to the amount of organic matter therein. The digestion of starch by the parotid secretion, after it reaches the stomach, is possible in proportion to the alkalinity of the saliva. The action of ptyalin in the stomach is only possible during the beginning and the final stages of digestion. At the acme of gastric digestion, when the free hydrochloric acid is abundant, the action of the saliva is only possible when there are large amounts of highly alkaline saliva in the stomach. The influence of saliva in the digestion of proteids reduces itself to a dilution of the hydrochloric acid of the gastric juice, and in disease makes hypoacidity worse, while it tends to make hyperacidity less severe.—E. A. Zherbovski (*Roussky Vratch,* March 5, 1905; *New York Medical Journal* and *Philadelphia Medical Journal,* April 29, 1905), *Cyclopedia of Practical Medicine.*

